# 2D Electrodes From Functionalized Graphene for Rapid Electrochemical Gold Extraction and Reduction From Electronic Waste

**DOI:** 10.1002/advs.202408533

**Published:** 2024-11-06

**Authors:** Kou Yang, Konstantin G. Nikolaev, Xiaolai Li, Ivan Erofeev, Utkur M. Mirsaidov, Vasyl G. Kravets, Alexander N. Grigorenko, Xueqing Qiu, Shanqing Zhang, Kostya S. Novoselov, Daria V. Andreeva

**Affiliations:** ^1^ School of Chemical Engineering and Light Industry Guangdong University of Technology Guangzhou 510006 China; ^2^ Institute for Functional Intelligent Materials National University of Singapore Singapore 117544 Singapore; ^3^ Department of Materials Science and Engineering National University of Singapore Singapore 117575 Singapore; ^4^ Department of Biological Sciences National University of Singapore Singapore 117558 Singapore; ^5^ Centre for BioImaging Sciences National University of Singapore Singapore 117543 Singapore; ^6^ Department of Physics National University of Singapore Singapore 117551 Singapore; ^7^ Department of Physics and Astronomy Manchester University Manchester M13 9PL UK

**Keywords:** chemisorption, chitosan, electrochemical reduction, electronic waste, gold extraction, graphene oxide, membrane

## Abstract

Electronic waste (e‐waste) contains substantial quantities of valuable precious metals, particularly gold (Au). However, inefficient metal recovery leads to these precious metals being discarded in landfills, causing significant water and environmental contamination. This study introduces a two‐dimensional (2D) electrode with a layered graphene oxide membrane functionalized by chitosan (GO/CS). The GO/CS membrane acts as an ion‐selective layer and demonstrates capabilities in the electrochemical extraction and reduction of Au ions. The multiple functional groups of GO and CS offer high cooperativity in ion extraction and reduction, achieving 95 wt.% extraction efficiency within 10 min. The simultaneous extraction and electrocatalytic reduction of Au ions within the membrane leads to the formation of ready‐to‐use metallic Au forms such as chips and sensors. Such an approach eliminates the processing steps required to convert extracted gold into functional products, reducing time, cost, and energy. This direct formation of usable Au components enhances the efficiency of the recovery process, making it economically viable and environmentally sustainable. The gold mining market is projected to be valued at $270 billion by 2032, with the recycling segment reaching $10.8 billion, highlighting the substantial benefits and economic potential of efficient e‐waste recycling technologies.

## Graphical abstract

This study presents a two‐dimensional (2D) electrode comprising a multilayered graphene oxide membrane functionalized with chitosan (GO/CS). The GO/CS membrane functions as an ion‐selective array of nanochannels, demonstrating effective chemical extraction and electrochemical reduction of gold ions. The multiple functional groups in GO and CS provide strong cooperativity, leading to an extraction efficiency of 95 wt.% within just 10 min.

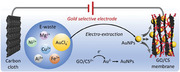

## 1 Introduction

Water pollution containing electronic waste (e‐waste) has been a global environmental issue for decades due to the indiscriminate disposal and mishandling of discarded electronic devices. E‐waste contains large amounts of precious metals that end up in landfills due to a lack of metal recovery measures. Gold (Au), one of the most valuable metals in e‐waste, urgently needs to be recycled from e‐waste to maintain its economic value. According to various market research consultancy reports, the gold mining market size was valued at ≈$200 billion in 2022, with a projected growth to ≈$270 billion by 2032 at a compound annual growth rate (CAGR) of 3.50%. Considering that the recycling segment comprises ≈4% of the market, this segment was valued at roughly $8 billion in 2022, with a projected growth of $10.8 billion by 2032 at the same CAGR. Therefore, scientists have been working to develop methods to extract precious metals from e‐waste such as adsorption,^[^ ^1^ ^]^ membrane filtration,^[^ ^2^ ^]^ ion exchange,^[^ ^3^ ^]^ reverse osmosis,^[^ ^4^ ^]^ and electrolysis^[^ ^5^ ^]^ techniques. Among them, adsorption is the most practical method because of its low cost and simplicity of operation. Many materials have been employed as efficient metal ion adsorbents for the purification of contaminated water. The most prevalent adsorbents are materials with high porosity, which are capable of capturing Au ions by their internal pores and chemically reducing them by a variety of functional groups.

High surface area, conductivity and available ion‐binding sites are essential for efficient metal adsorbents. Among carbon‐based materials,^[^ ^6^ ^]^ graphene oxide (GO) is considered a potential high‐capacity metal ion adsorbent, due to its high surface area and tunable conductivity.^[^ ^7^ ^]^ Mechanical flexibility, chemical stability, and compatibility with polymers make it a promising candidate for water purification adsorbents.^[^ ^8^ ^]^ GO possesses a large number of oxygen‐containing functional groups, such as epoxy, hydroxyl, and carboxyl groups, which are capable of binding metal cations through electrostatic interactions.^[^ ^9^ ^]^ Ion‐binding sites, known as chelating functional groups, such as hydroxyl and amino groups are significantly reactive to metal ions.^[^ ^10^ ^]^ Chitosan (CS) molecules contain such ion‐binding sites and can function as a sponge to attract and extract ions. Furthermore, intrinsic functional groups of chitosan and other polyamines, starch‐based biomaterials,^[^ ^11^ ^]^ diamides,^[^ ^12^ ^]^ and cyclodextrins^[^ ^13^ ^]^ promote the reduction of metal ions to form metal nanoparticles. In terms of sustainability and economic impact, CS is an efficient bio‐sorbent for metal extraction. CS is a highly abundant polymer from natural sources.^[^ ^14^ ^]^ Here, we assembled layered CS with GO to form a composite membrane with high surface area, tunable conductivity, and ion‐binding sites to maximize the ion extraction efficiency of both compounds.

The GO and CS are both employed for the extraction of ions. CS exhibits a strong affinity for chelation with metal ions, particularly those of transition metals. Similarly, GO can recover metal ions from heavy metal waste, attributed to its negatively charged functional groups and the expansive specific surface area of its 2D flakes. In this study, we strategically combine the ion adsorption capacities of both GO and CS, resulting in the creation of highly efficient composites for the recovery and reduction of Au ions. By constructing multi‐layered composite membranes with CS assembled on 2D GO flakes, we significantly increase the number of functional interfaces available for ion adsorption. This approach not only amplifies the ion adsorption capacity of both materials but also optimizes the ion extraction yield. Consequently, by adjusting the surface chemistry and surface area of our composites, we have achieved maximum efficiency in Au recovery.

## 2 Results and Discussion

**Figure 1:**
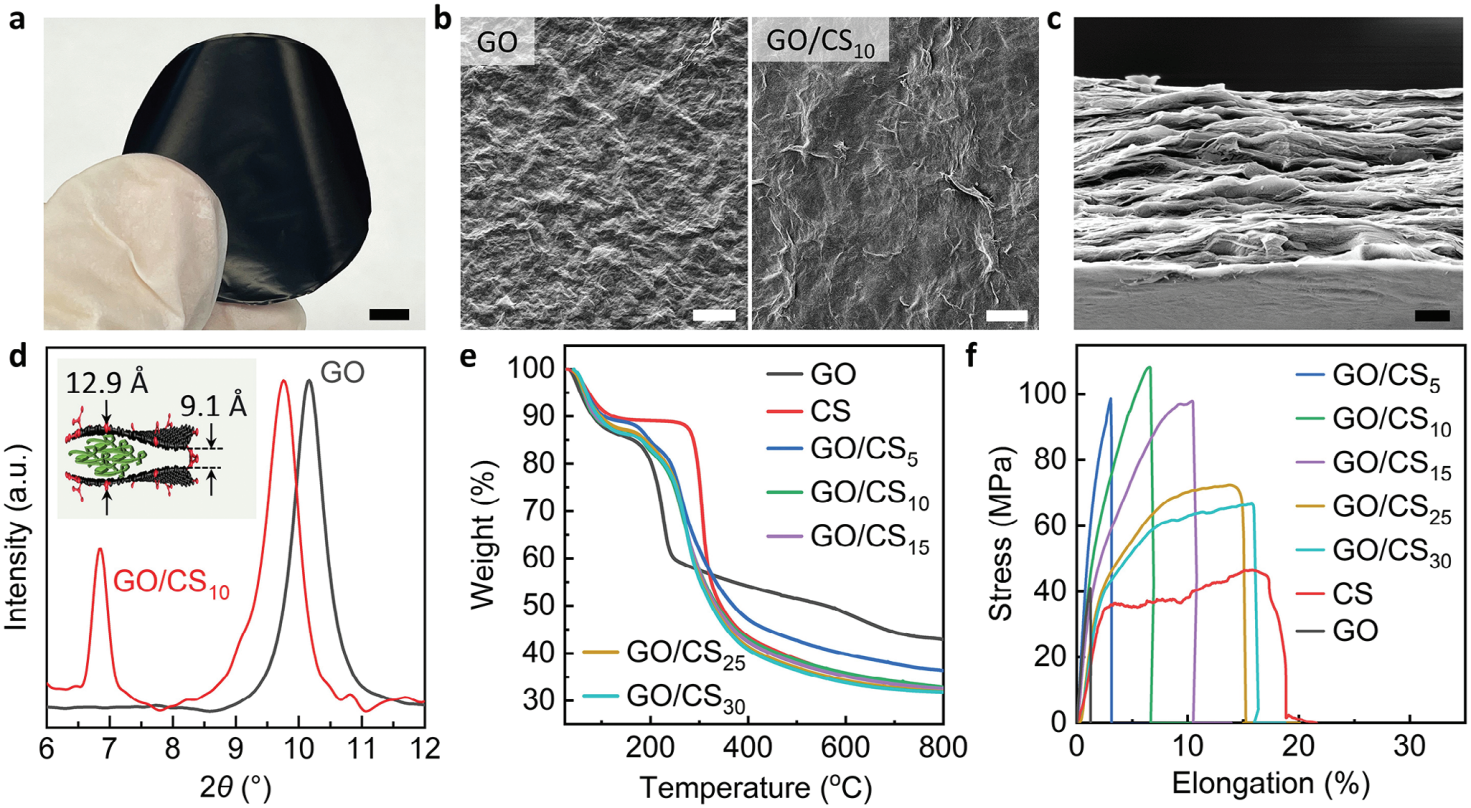
Characterization of GO/CS membranes. a), Photograph of a free‐standing GO/CS_10_ membrane with 5 mg GO loaded, scale bar: 5 mm. b), Scanning electron microscopy (SEM) images of a 5 mg pristine GO membrane (left) and GO/CS_10_ membrane (right), scale bars: 10 µm. c), Cross‐sectional SEM image of a free‐standing GO/CS_10_ membrane with 5 mg GO loaded, scale bar: 1 µm. d), XRD patterns of GO and GO/CS_10_. e), TGA curves of GO, CS, and GO/CS composite membranes. Heating rate: 10 °C min^−1^, in a nitrogen atmosphere. f), Stress‐strain curves of pristine GO membrane, pristine chitosan membrane, and GO/CS composite membranes. The composite membranes are designated as GO/CS_x_, where x = 5, 10, 15, 25, 30, representing the mass ratio of CS to GO.

Ion‐selective GO/CS membranes are fabricated through the self‐assembly of GO nanosheets and CS macromolecules (see Experimental Section for details). Initially, CS is dissolved in 1% v/v acetic acid (HOAc) and magnetically stirred at ambient temperature for a duration of 48 h, resulting in a stable CS/HOAc dispersion. The subsequent step involves the mixture of the CS dispersion with the GO dispersion at a specific mass ratio, forming a homogeneous composite dispersion. The composite membranes are designated as GO/CS_x_, where x = 5, 10, 15, 25, 30, representing the mass ratio of CS to GO. Within this dispersion, GO and CS are bound by electrostatic forces, where the positively charged NH^3+^ groups on chitosan interact with the negatively charged COO‐ groups on GO. As depicted in **Figure** **1**a, a robust 2D GO/CS membrane can be prepared via vacuum filtration. The thickness of such membranes varies from tens of nanometres to microns depending on the amount of used GO/CS. The specific surface area can be calculated for the known amount of GO and CS by mass and estimated surface area.^[^ ^15^ ^]^ In this study, the 0.1 mg GO in the final GO/CS composite sample corresponds to a surface area of 0.26 m^2^. It is important to note that all the chemical methods employed in this study are environmentally friendly and sustainable. In fact, the concentration of acetic acid in the GO/CS composite dispersion is even lower than that found in edible vinegar.

The GO/CS membrane exhibits a typical surface and layered cross‐sectional structure of a pristine GO membrane (Figure 1b,c; Figure S1, Supporting Information). GO nanosheets and CS macromolecules are perfectly aligned to form a layer‐by‐layer structure. The X‐ray diffraction (XRD) analysis reveals the existence of two distinct interlayer distances within the membrane, indicating the effective encapsulation of chitosan molecules between the GO laminates (Figure 1d). The interlayer spacing (*d*) of CS encapsulated between the GO laminates is 12.9 Å, while for the region without CS, *d* is 9.1 Å. Importantly, the thermogravimetric analysis (TGA) (Figure 1e) and mechanical tests (Figure 1f) revealed that the GO/CS_10_ has an optimal set of thermal and mechanical stability. The entrapment of CS molecules within the 2D confinement of GO leads to a significant improvement in the membrane's mechanical properties, with the ultimate tensile strength and Young's modulus of the GO/CS_10_ membrane (6 GPa) exceeding those of both pure GO and CS. Beyond this ratio, the excess CS forms a bulk phase that doesn't improve the stability of the material (Figure 1f). These advanced structural and functional characteristics of the GO/CS membrane provide a solid foundation for further optimization and real‐world applications in the field of gold recovery from e‐waste.

**Figure 2:**
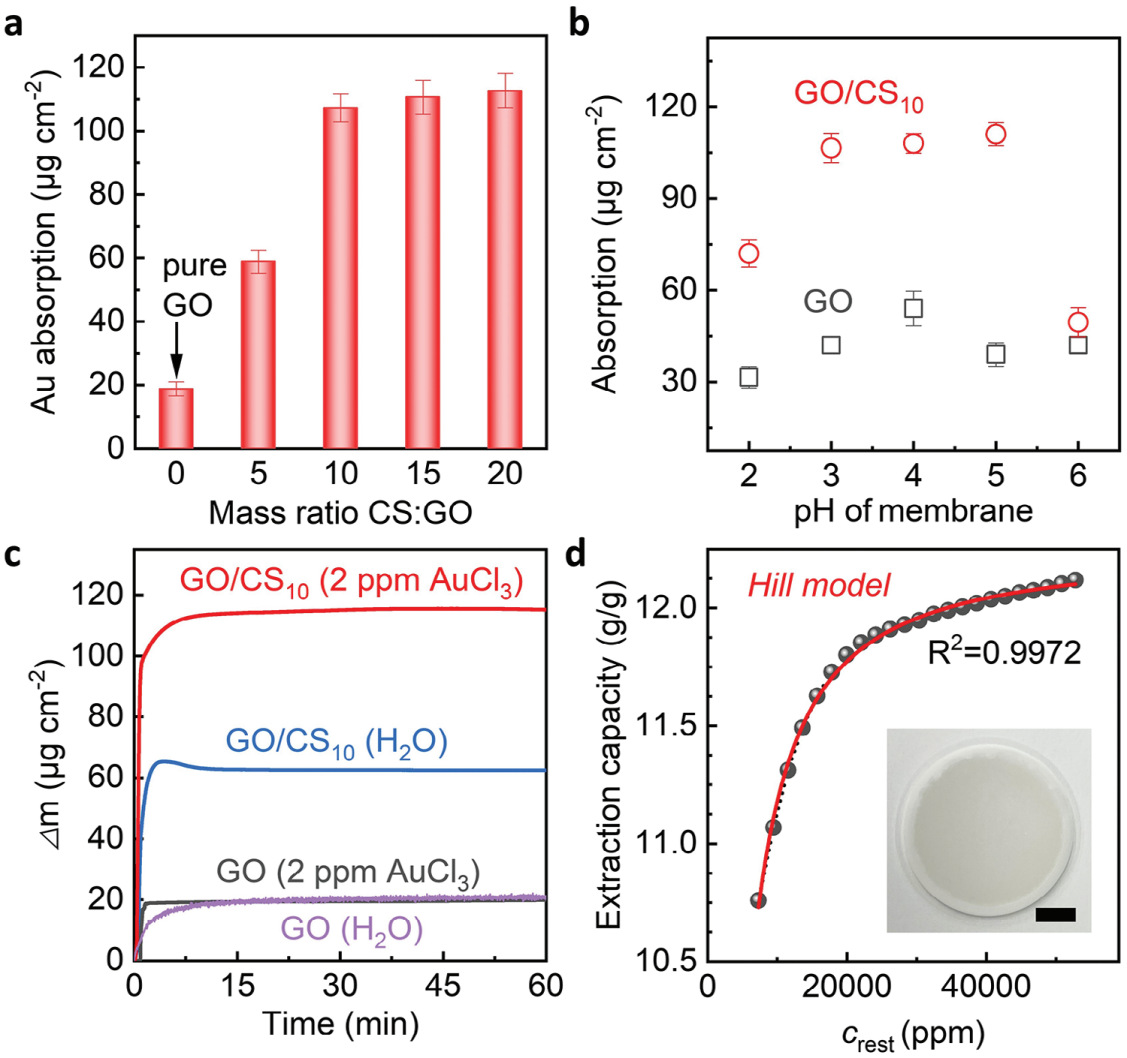
Gold extraction properties of GO/CS membranes. a,b, Au concentration inside GO and GO/CS membranes as a function of CS: GO mass ratio (a) and the pH of membranes after 1 h permeation process, 2 ppm of AuCl_3_ is used as feed solution. c, QCM curves showing the mass change of GO and GO/CS_10_ in 2 ppm AuCl_3_ or water versus time. d, Extraction capacity of GO/CS_10_ composite membrane calculated from QCM measurement, 2 ppm of AuCl_3_ is used as feed solution. Inset: photograph showing a 75 nm GO/CS_10_ membrane prepared for QCM measurements, scale bar: 1 cm.

The layered structure of GO provides a substantial surface area, which is further enhanced by the presence of functional groups in CS that facilitate additional interactions with metal ions. The synergistic combination of GO and CS significantly enhances the adsorption of Au^3+^, surpassing the capabilities of pristine GO membranes, as illustrated in **Figure** **2**a,b. To ascertain the optimal composition for Au adsorption, experiments were conducted using an aqueous Au^3+^ solution at a concentration of 2 ppm. The concentrations of gold in the solution before and after adsorption were quantified using inductively coupled plasma‐optical emission spectroscopy (ICP‐OES), with further methodological details provided in the Experimental Section. Notably, the GO/CS membranes demonstrated a saturation point in Au^3+^ adsorption upon reaching a mass ratio of 10 for GO and CS (Figure 2a). This saturation suggests that the gold binding sites within the GO/CS membrane are fully occupied, aligning with the results obtained from the earlier TGA and mechanical analyses. This evidence underscores the effectiveness of the GO/CS composite membranes in maximizing gold ion capture, thereby optimizing the material's design for enhanced performance in metal ion extraction from electronic waste.

The peak adsorption observed in the pH range of 3–5, which falls in the pKa window of GO (pKa 4.3) and CS (pKa 6.5), can be attributed to several synergistic factors. First, this pH range is optimal for maintaining a significant degree of protonation of chitosan's amino groups while avoiding excessive charge repulsion. This balance maximizes electrostatic attractions between negatively charged sites on the GO and the positively charged chitosan, thereby enhancing the overall binding capacity for Au^3+^. On the other hand, the negatively charged carboxyl groups on GO edges interact with the protonated amino groups of chitosan and/or directly with the metal ions, providing multiple binding sites that are chemically diverse and spatially optimized for interaction with Au^3+^.

To assess the extraction rate and equilibrium time for gold ions, we utilized the dynamic quartz crystal microbalance (QCM) technique to monitor the adsorption kinetics of GO/CS composite membranes. This method allowed us to precisely measure the changes in mass corresponding to the adsorption of Au^3+^ over time (see Experimental Section for a comprehensive description of QCM setup). As shown in Figure 2c and Figure S2 (Supporting Information), the GO/CS_10_ membrane demonstrated an adsorption efficiency, capturing up to 95 wt.% of Au^3+^ within the first 10 min of exposure at room temperature, across all tested concentrations of Au^3+^. This adsorption rate is significantly faster than that observed with other graphene‐based adsorbents, which typically require several days to achieve maximum Au extraction levels. The extraction efficiency of the GO/CS composite membranes, in comparison to other materials,^[^ ^11^, ^16^ ^]^ is provided in Table S1 (Supporting Information). The extraction capacity of the GO/CS composite membranes is several times higher than that of rGO^[^ ^16g^ ^]^ and other 2D materials^[^ ^16c,i^ ^]^ a few times higher. The S‐and N‐containing polymers^[^ ^11^, ^16^ ^]^ exhibit extraction capacities that are even ten times lower.

Fitting of the QCM data using the Hill model (Figure 2d) demonstrates the highest correlation coefficient compared to the fitting parameters obtained from Langmuir, Freundlich and Temkin isotherms (Figure S3, Supporting Information).

Importantly, in our case the Hill equation results in a typical sigmoidal curve when Au ions extraction capacity is plotted against Au ions feed concentration:

(1) $$q_{e}=\frac{q_{max}\cdot C_{rest}^{n}}{K^{n}+C_{rest}^{n}}$$

where *q_e_* is the equilibrium adsorption capacity, *q_max_* – maximal theoretical adsorption capacitance, *C_rest_* – rest concentration after adsorption, 1*/K* is the adsorption constant (*K_ads_*), *n* – is the stochiometric coefficient for the number of ligands that bind with molecules.

The Hill coefficient (*n*) represents the degree of cooperativity. If n is greater than 1, it indicates positive cooperativity (binding of one ligand enhances the binding of subsequent ligands). The positive cooperativity in the formation of CS‐NH_2_∙∙∙Au^3+^∙∙∙HOOC‐GO complexes leads to a nonstoichiometric interaction between Au^3+^ and the adsorption centers of the GO/CS membrane. As a result, the subsequent electroreduction of Au^3+^ occurs effectively, unhindered by restrictions from the preceding adsorption step. From the Hill isotherm model for GO/CS membrane, we found that the n value is 1.49. Thus, the study of kinetics reveals an ion adsorption phenomenon that suggests a typical biological macromolecule cooperativity, where the binding of an ion at one site affects the binding of ions at other sites on the same macromolecule. Additionally, a value of *R* ^2^ of 0.9999 was calculated with the pseudo‐second‐order model (Figure S4, Supporting Information). Furthermore, we calculated the rate constants for the adsorption on the GO/CS membrane to be 0.29 g/g∙min. The rapid and efficient kinetics of Au ion extraction observed in our material arise due to the capillary action in the nanochannels with heterogeneous surface chemistry, featuring hydrophobic sp^2^ carbon domains and acetyl groups (─COCH_3_) as well as multiple hydrophilic groups.

**Figure 3:**
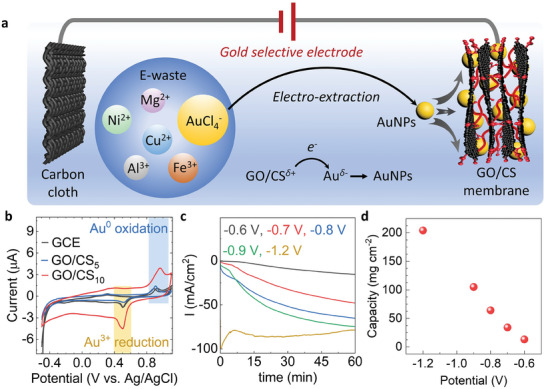
Electrochemical gold extraction assisted by GO/CS membrane. a, Schematic diagram of GO/CS membrane using as an Au selective layer for the formation of Au selective electrode for electro‐extraction and reduction of Au, *δ* = 1 or 3. b, Cyclic voltammetry curves of bare glassy carbon electrode (GCE), GO/CS_5_ and GO/CS_10_ membrane electrodes at a scan rate of 10 mV s^−1^ in 0.1 m HCl solution with Ar purged. The concentrations of both Au^3+^ and Cu^2+^ are 200 ppm. c, I–t curves when applying different negative potentials on GO/CS_10_ membrane immersed in 2 ppm of Au^3+^ in 0.1 m HCl for 1 h. d, Au extraction capacity as a function of applied potential.

The GO/CS membrane serves as an Au‐selective layer on the electrode surface for electrochemical ion recovery and reduction (**Figure** **3**a). The Au‐selective electrode is capable of extracting ions from the e‐waste mixed ions solution under applied voltage, while also simultaneously reducing them to form metallic forms. The Au ions extraction experiments were performed in a three‐electrode system. A carbon cloth (CC) with a GO/CS membrane serves as the working electrode, and pure CC and Ag/AgCl (in 3 m KCl electrolyte solution) are used as counter and reference electrodes, respectively (Figure S5, Supporting Information).

Extraction kinetics experiments were performed at a constant voltage of −1.2 V (relative to Ag/AgCl)^[^ ^17^ ^]^ in an acidic mixture (25 mL) containing 20 ppm or 200 ppm of Au^3+^/ Cu^2+^. From the cyclic voltammogram (CV) profiles of the GO/CS membrane and bare glassy carbon electrode (GCE), nonsignificant shifting of oxidation peaks of Au^0^ and reduction peaks of Au^3+^ can be observed (Figure 3b). Therefore, the Au redox equilibrium potential changed from 0.70 V, for the bare GCE, to 0.73 V for the GO/CS_10_. The current density values drastically increased for the GO/CS_10_ in comparison with bare GCE. Such negligible change of the equilibrium potential and increased current density proves that the GO/CS membrane does not affect the charge transfer resistance and can be utilized for the electrowinning process. The specific capacitance value of the GO/CS membrane electrode is enhanced in comparison with bare GCE.

As expected, GO/CS_10_ is more effective for Au extraction compared to GO/CS_5_, aligning with previous findings on the extraction properties of GO/CS membranes, further illustrating the tunable nature of this composite material for specific application needs. Additionally, Furthermore, the GO/CS membrane's efficiency in Au extraction was found to be dependent on the applied voltage, as illustrated in Figure 3c,d. Increasing the voltage linearly enhances the extraction capacity, likely due to the accelerated electro‐diffusion of Au ions into the membrane followed by their electro‐reduction. This enhancement in performance with voltage underscores the membrane's potential for optimized Au recovery in practical applications.

**Figure 4:**
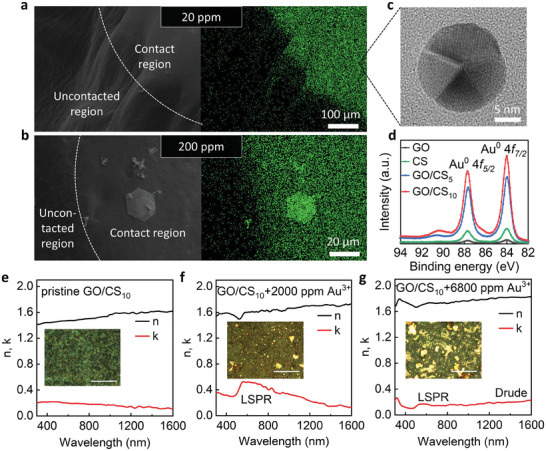
AuNPs formation and spectroscopic ellipsometry of GO/CS membranes. a,b, SEM image (left) and EDX mapping (right) of GO/CS_10_ membrane after gold extraction in 20 ppm (a) and 200 ppm (b) of Au^3+^/Cu^2+^ mixture, the white dashed line indicates the border of contact and uncontacted region. c, TEM image of AuNPs deposited on GO/CS_5_ membrane after electro‐extraction in 20 ppm Au^3+^/Cu^2+^ mixture. d, 4*f* XPS spectrum of gold nanoparticles on pristine GO, CS and GO/CS membranes after electro‐extraction of Au^3+^. e–g, Extracted optical constants of a pristine GO/CS_10_ membrane (e), GO/CS_10_ membrane subject to 2000 ppm of Au^3+^ (f) and GO/CS_10_ membrane subject to 6800 ppm of Au^3+^ (g). Insets: optical images of the tested membranes. “LSPR” denotes the position of localized plasmon surface resonances of gold nanoparticles. “Drude” indicates the position of the Drude response of the sample. White scale bars: 50 µm.

**Figure** **4** is a detailed examination of gold nanoparticles (AuNPs) generated on GO/CS composite membranes following the electrochemical extraction and reduction of Au from solutions containing Au ions. The SEM images alongside energy dispersive X‐ray spectroscopy (EDX) mappings of the GO/CS_10_ membrane reveal the surface morphology of the membranes after being treated with Au ion solutions at concentrations of 20 and 200 ppm, respectively (Figure 4a,b). The SEM images, show distinct regions where the membranes were in contact with the Au solutions, demarcated by white dashed lines. The corresponding EDX maps, with the Au element highlighted in green, show the spatial distribution of the synthesized AuNPs. Notably, the density of these NPs is visibly higher in the 200 ppm treatment compared to the 20 ppm, indicating a concentration‐dependent loading of AuNPs on the membrane. For the e‐waste containing 200 ppm of Au^3+^, monocrystalline AuNPs are observed on the GO/CS membrane (Figure 4b).

Due to the deposition of Au, the GO/CS surface changed from black to gold (see the photo inserted in Figure S5, Supporting Information), indicating that a substantial amount of Au^0^ was successfully electro‐extracted and reduced from the simulated e‐waste solution and embedded in the membrane. The observation of lattice structure and grain boundaries of AuNPs in the transmission electron microscopy (TEM) image (Figure 4c) provides insights into the nano‐scale morphology and confirms the successful reduction of gold ions to elemental gold on the membrane surface. Moreover, the appearance of the 4*f* peaks of Au^0^ in the X‐ray photoelectron spectroscopy (XPS) data (Figure 4d) further supports the electro‐reduction of Au^3+^ to AuNPs with a size smaller than 20 nm. As a result, our membrane exhibits selective extraction of Au ions under an applied voltage and can also serve as a template for the formation of AuNPs.

In addition to the electrochemical extraction of gold from e‐waste, the GO/CS membrane embedded with metallic gold is a ready‐to‐use optical sensor. To investigate the distribution of gold inclusions extracted by GO/CS membranes, we performed variable angle spectroscopic ellipsometry measurements on samples that were subject to different Au concentrations. Spectroscopic ellipsometry records optical spectra with higher accuracy than absorption spectroscopy and allows one to extract the optical constants of the studied structures (see Supporting Information for details).

Representative ellipsometry spectra of GO/CS samples subject to the solutions of different Au concentrations are shown in Figure 4e,f. Combined with the ellipsometry phase ∆ (Figure S6, Supporting Information), these spectra allowed us to retrieve the optical constants of the samples. Figure 4e shows the extracted optical constants (*n* and *k*) for a GO/CS matrix. We see that the spectra of the optical constants are reasonably flat in the studied range of 300–1600 nm for pristine GO/CS_10_ samples. After being subject to an Au concentration of 2000 ppm, a GO/CS_10_ membrane extracted Au in the form of AuNPs of different shapes and sizes. As a result, the localized surface plasmon resonance (LSPR) of these AuNPs can be clearly seen as a peak in the absorption coefficient *k* in the extracted optical constants of GO/GS samples subject to low concertation of Au (Figure 4f). This LSPR peak is quite wide and shifted to red wavelengths as compared to pristine Au. Since both the shape and size of the AuNPs (as well as the matrix environment) can affect the plasmon resonances,^[^ ^18^ ^]^ it is difficult to assign the widening of the LSPR peak to either the shape or size of AuNPs. From the inset of Figure 4f, we can assume that both factors are important. At higher Au concentrations, AuNPs extracted with the help of a GO/GS membrane could also produce a connected network which normally results in the Drude behavior associated with the metal response at infrared wavelengths. Hence, for a GO/GS sample subject to 6800 ppm of Au concentration, we see both the LSPR at ≈600 nm wavelengths and the Drude response at the infrared range (*n* and *k* rising with increasing wavelengths) see Figure 4g.

## 3 Conclusion

This study introduces a novel layered GO/CS membrane, designed as an ion‐selective layer for 2D electrodes, to achieve rapid and efficient extraction of Au ions from e‐waste. The cooperativity between the functional groups of GO and CS enhances the membrane's ability to extract and reduce Au ions effectively. Detailed characterization has demonstrated the membrane's robust layered structure, as well as its excellent thermal and mechanical stability. The GO/CS membrane not only acts as an ion‐selective layer but also facilitates the electrocatalytic reduction of Au ions within its structure, resulting in the formation of ready‐to‐use metallic gold forms such as chips or sensors. This capability is significant because it removes the need for additional processing steps to convert extracted Au into usable products, thereby reducing time, cost, and energy consumption. This direct formation of functional gold components significantly enhances the overall efficiency of the recovery process, making it both economically viable and environmentally sustainable. By addressing the environmental impacts of improper e‐waste disposal, the GO/CS membrane not only improves Au recovery efficiency but also marks a substantial advancement in developing sustainable methods for e‐waste management and resource conservation. The significance of novel materials for ions extraction from e‐waste lies in their potential to revolutionize the way we maintain natural resources. By embracing circular economy principles, enhancing sustainability, and integrating simultaneous extraction and reduction processes, these materials provide a holistic solution to the challenges of resource recovery and environmental conservation. Their implementation not only promotes the responsible management of electronic waste but also drives innovation toward a more sustainable and economically viable future. The implementation of novel materials for Au extraction from e‐waste offers significant benefits, including reducing e‐waste in landfills by up to 50%, cutting energy consumption in recovery processes by 40%, and increasing the economic value of recovered materials by 30%.

## 4 Experimental Section

### Materials

Aqueous graphene oxide dispersion (GO, 4 mg mL^−1^, monolayer content >95%, Graphenea Inc., USA). Chitosan (CS, powder, from shrimp shells, approx. Mw = 190–375 kDa, Sigma–Aldrich). Acetic acid (HOAc, glacial, ReagentPlus, ≥99%, Sigma–Aldrich). Hydrochloric acid (HCl, ACS reagent, 37%, Sigma–Aldrich). Sodium hydroxide solution (NaOH, 50% in H2O, Sigma–Aldrich). Gold (III) chloride (AuCl_3_, powder, catalyst reagent type, 99%, Sigma–Aldrich). Copper (II) Chloride (CuCl_2_, powder, 99%, Sigma–Aldrich). Polyethersulfon membrane filter (PES, 0.03 µm, 47 mm, Sterlitech Corporation, USA), Anodisc 47 filter (pore size – 0.02 µm, diameter 47 mm, Whatman, USA). All materials were received and used without further purification.

### Preparation of GO/CS Composite Membranes

The original aqueous graphene oxide dispersion (4 mg mL^−1^, 20 mL) was added into deionized water (380 mL) to obtain diluted GO dispersion (0.2 mg mL^−1^). CS/HOAc dispersion (5 mg mL^−1^) was obtained by dissolving chitosan (2 g) in HOAc (1 vol%/vol, 400 mL) upon magnetic stirring for 48 h at room temperature. Then, CS/HOAc dispersion was mixed with GO dispersion (volume varies according to different mass ratios of GO and CS), and the colloids were then mixed for 10 min by a shaker (rotation speed – 500 rpm, Vortex Mixer, USA). GO/CS composite membranes were prepared by vacuum filtration of the aforesaid mixture through two types of membrane filters: Anodisc 47 and polyethersulfone membrane. Vacuum filtration was maintained for 24 h, and the obtained membrane was then dried overnight in a dry cabinet at room temperature. For comparison, a pristine GO membrane can be easily prepared by vacuum filtrating GO dispersion through the filter.

### Setup for Gold Adsorption Tests

Experiments were performed using a set of side‐by‐side diffusion cells (Yuyan Instruments, Shanghai) with a pristine GO membrane or GO/CS membrane (with 5 mg of GO loading) fixed between two cell compartments, for example, the feed compartment and the drain compartment. The feed compartment was filled with AuCl_3_ solution (20 mL), while the drain compartment was sucrose (20 mL, 2.5 mol L^−1^) to induce the osmotic pressure between the two cell compartments. After 1 h, the gold concentration inside the membrane was evaluated using Inductively Coupled Plasma‐Optical Emission Spectroscopy (ICP‐OES). Before elemental analysis, the solid membrane samples were digested with HNO_3_/HCl (3:1) in a microwave at 240 °C for 15 min and topped up to 10 mL with H_2_O. Note that a clear solution was observed before analysis.

### Preparation of GO/CS Membranes for QCM Measurements

CS/HOAc (40 µL, 5 mg mL^−1^) dispersion was mixed with GO dispersion (0.1 mL, 0.2 mg mL^−1^) and 5 mL of DI‐water, and the colloids were then mixed for 10 min by a shaker (rotation speed – 500 rpm, Vortex Mixer, USA). GO/CS thin film was prepared by vacuum filtration of the aforesaid mixture through an Anodisc 47 membrane filter. Vacuum filtration was maintained for 2 h, and the obtained film was then dried overnight in a dry cabinet at room temperature. Next, the prepared membrane was peeled off on the water's surface and transferred onto the surface of a 5 MHz Au electrode. QCM measurements were performed by the QSense Explorer System (QE 401 Electronic Unit, QCP 101 Chamber Platform, QFM 401 Flow Module). DI‐water or 2 ppm AuCl_3_ solution was pumped into the chamber at the speed of 50 uL min^−1^.

### Characterization Methods

X‐ray diffraction (XRD) was carried out using Bruker D8 ADVANCE with a Cu Kα tube radiation source (1.5418 Å). Thermogravimetric analysis (TGA) was performed by TA Instrument Discovery TGA1‐0247 under nitrogen at a heating rate of 10 °C min^−1^. The mechanical properties were measured using a dynamic mechanical analyser (DMA 850, TA Instruments). Scanning electron microscopy (SEM) images were obtained by a ZEISS Sigma 300 FE SEM system with EDX equipped. Before observation, the membranes were sputtered with 10 nm gold or carbon. Transmission electron microscopy (TEM) was conducted by a JEOL JEM‐2200FS electron microscope (JEOL Ltd., Tokyo, Japan) at 200 kV, equipped with a Direct Electron DE‐16 camera (Direct Electron, LP, San Diego, CA, USA.). The concentration of gold was measured by a Perkin Elmer Avio 500 inductively coupled plasma‐optical emission spectrometer (ICP‐OES). Quartz‐crystal microbalance (QCM) measurements were performed by the QSense explorer system (QE 401 Electronic Unit, QCP 101 chamber platform, QFM 401 flow module). Electrochemical measurements were conducted using BioLogic SP‐300 potentiostat. X‐ray photoelectron spectroscopy (XPS) was measured by Kratos AXIS UltraDLD.

## Conflict of Interest

The authors declare no conflict of interest.

## Supporting information



Supporting Information

## Data Availability

The data that support the findings of this study are available from the corresponding author upon reasonable request.
