# Supplementary material for: 2D Electrodes From Functionalized Graphene for Rapid Electrochemical Gold Extraction and Reduction From Electronic Waste
Source: Adv Sci (Weinh). 2024 Nov 6;12(1):2408533. doi: 10.1002/advs.202408533 (PMC11714188; doi:10.1002/advs.202408533)
Supplement: Supplementary file 1 — Supporting Information [file ADVS-12-2408533-s001.docx]

**Two-dimensional electrodes from functionalized graphene for rapid electrochemical gold extraction and reduction from electronic waste**

Kou Yang^1,2^, Konstantin G. Nikolaev^2^, Xiaolai Li^3^, Ivan Erofeev^4,5^, Utkur M. Mirsaidov^5,6^, Vasyl G. Kravets^7^, Alexander N. Grigorenko^7^, Xueqing Qiu^1^, Shanqing Zhang^1^, Kostya S. Novoselov^2,3^ and Daria V. Andreeva^2,3^*

*^1^School of Chemical Engineering and Light Industry, Guangdong University of Technology, 510006, Guangzhou, China*

*^2^Institute for Functional Intelligent Materials, National University of Singapore, 117544, Singapore*

*^3^Department of Materials Science and Engineering, National University of Singapore, 117575, Singapore*

*^4^Department of Biological Sciences, National University of Singapore, 117558, Singapore*

*^5^Centre for BioImaging Sciences, National University of Singapore, 117543, Singapore*

*^6^Department of Physics, National University of Singapore, 117551, Singapore*

*^7^Department of Physics and Astronomy, Manchester University, Manchester M13 9PL, United Kingdom*

Contents

1. Supplementary Fig.1 Cross-sectional SEM image of GO/CS_15._
2. Supplementary Fig.2 QCM curves of GO and GO/CS_10_ membranes.
3. Supplementary Fig.3 Isotherm models fitted for the GO/CS_10_ membrane adsorption capacity.
4. Supplementary Fig.4 Kinetic fitting results of pseudo-first and pseudo-second order of reaction for GO/CS membrane (for 200 ppm Au^3+^).
5. Supplementary Fig.5 Schematic illustration of the electrochemical system for the separation and recovery of gold from Au^3+^/Cu^2+^ mixture.
6. Supplementary Fig.6 The amplitude spectroscopic parameter of GO/CS membranes.


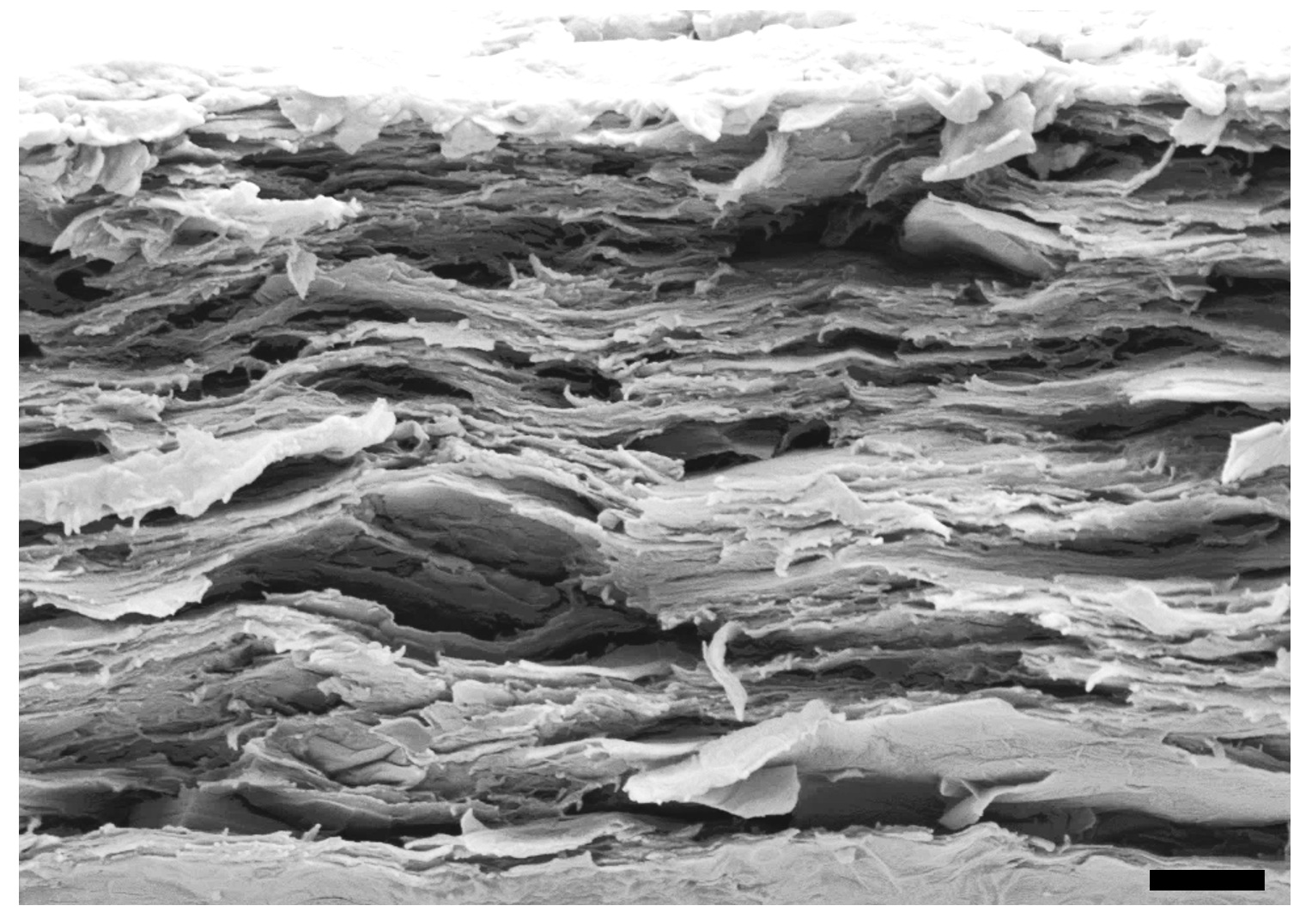


**Supplementary Fig.1** Cross-sectional SEM image of a free-standing GO/CS_10_ membrane with 5 mg GO loaded, scale bar: 1 μm.


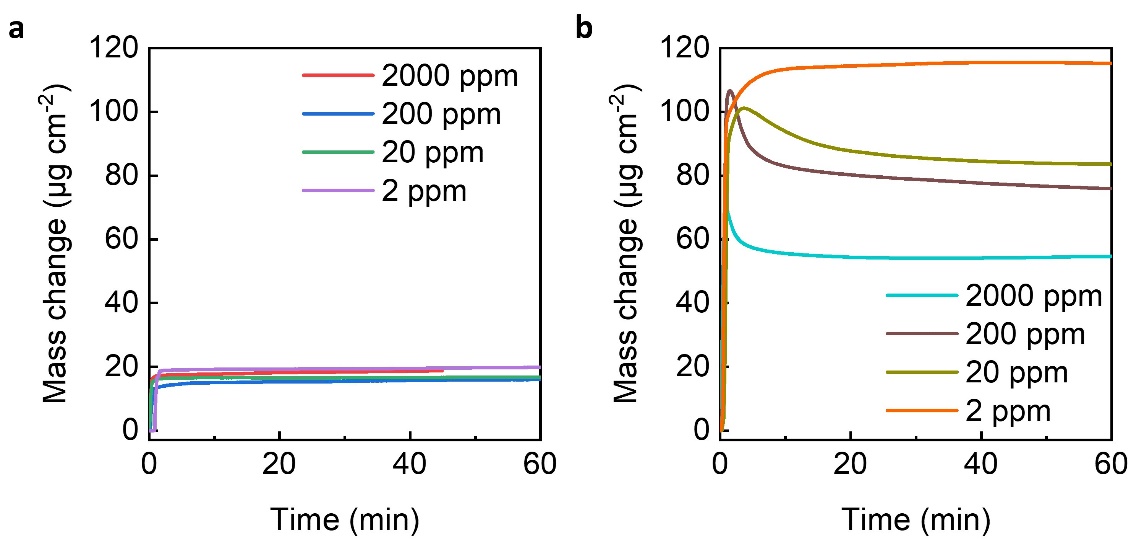


**Supplementary Fig.2** QCM curves showing the mass change of GO (a) and GO/CS_10_ (b) in different concentrations of AuCl_3_ or vs. time.


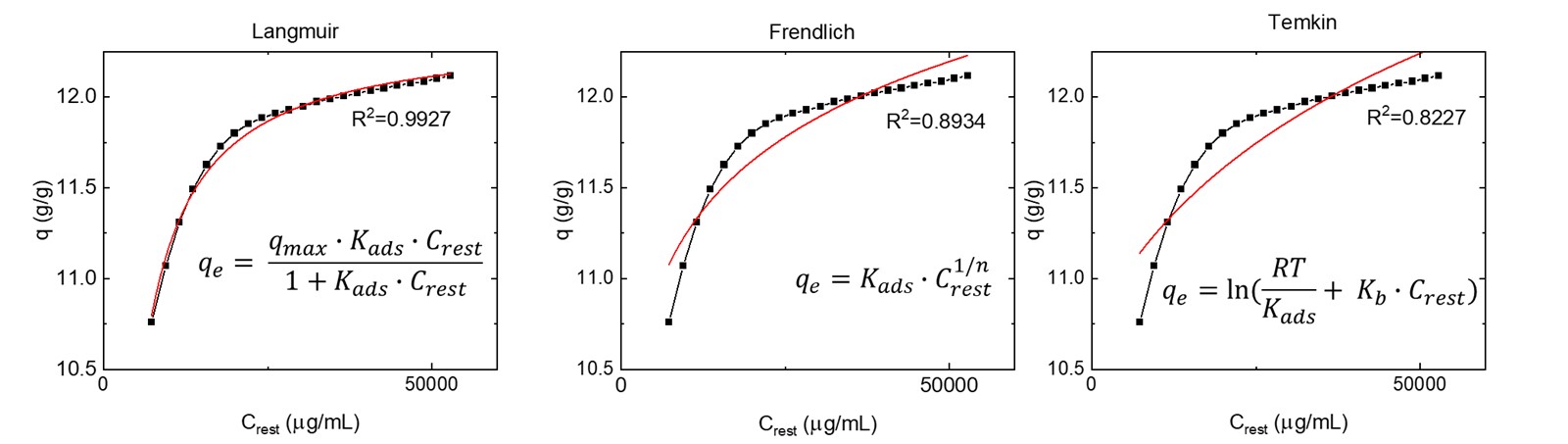


**Supplementary Fig.3** Isotherm models fitted for the GO/CS_10_ membrane adsorption capacity.


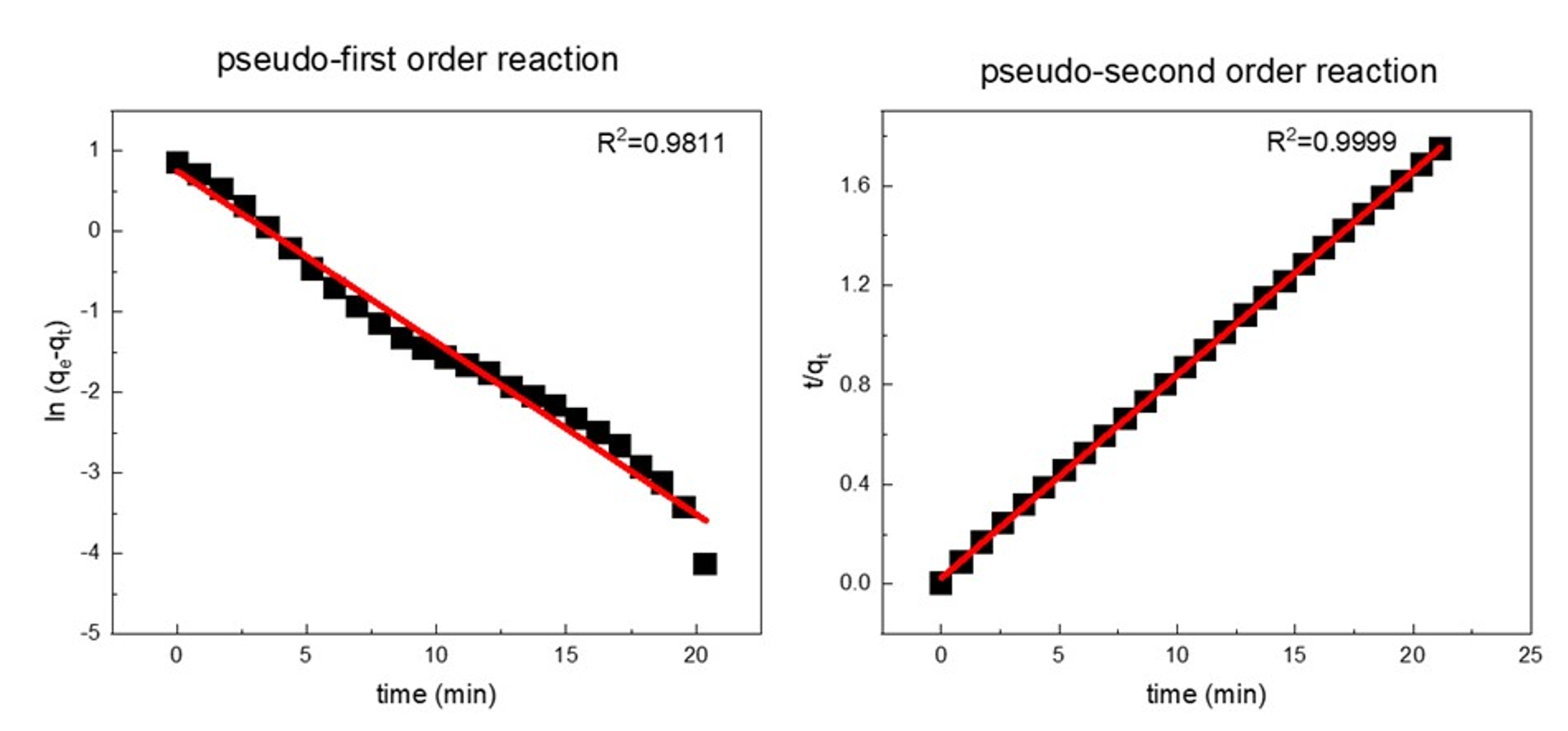


**Supplementary Fig.4** Kinetic fitting results of pseudo-first and pseudo-second order of reaction for GO/CS membrane (for 200 ppm Au^3+^).


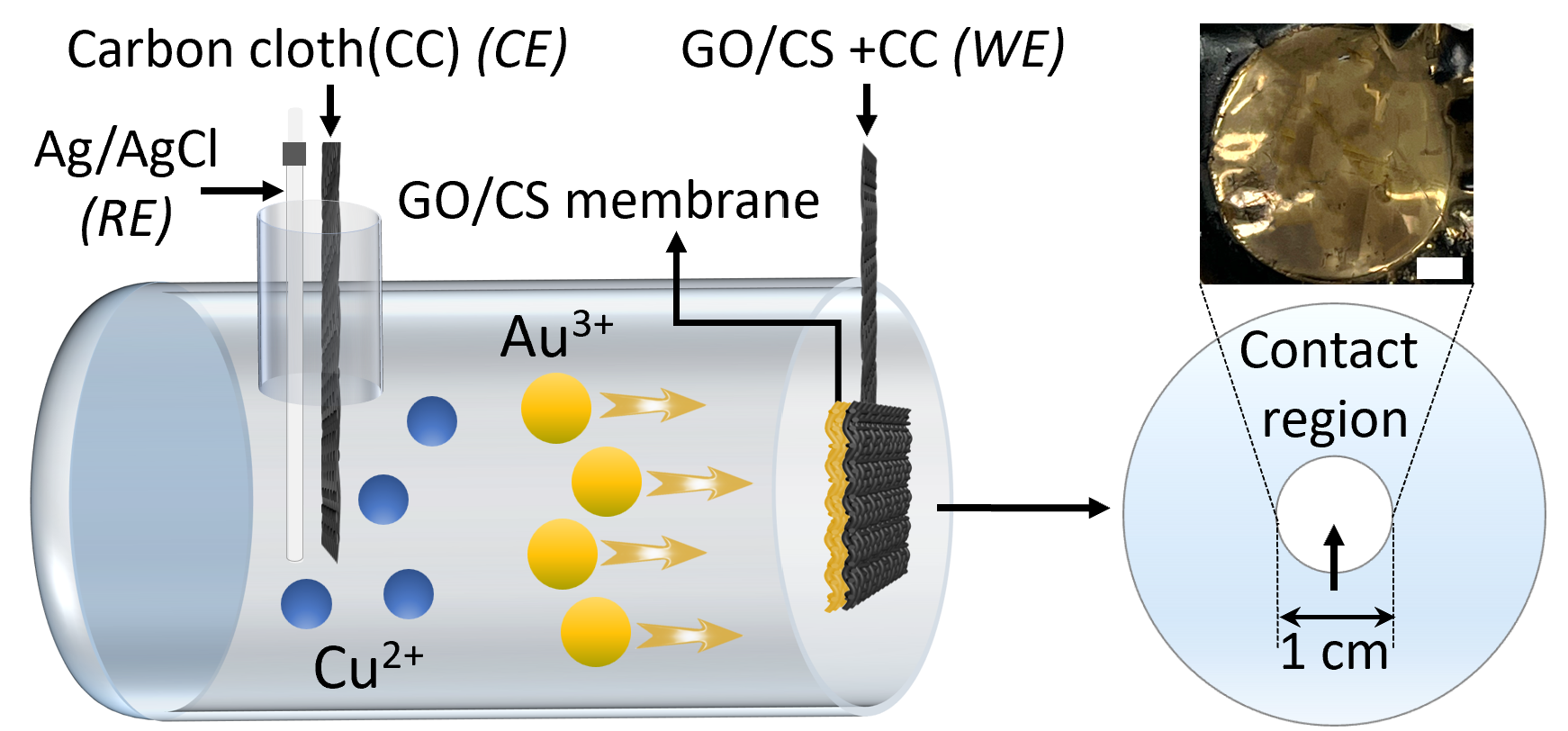


**Supplementary Fig.5** Schematic illustration of the carbon cloth // GO/CS_10_ membrane electrochemical system for the separation and recovery of gold from Au^3+^/Cu^2+^ mixture. A 1 cm diameter window on one side of the electrolyte container allows the contact between the electrolyte and the GO/CS membrane attached to the external wall of the container. We use this window to distinguish between gold-containing membranes and gold-free parts during cycles.

**Spectroscopic ellipsometry of GO/CS membranes**

Spectroscopic ellipsometry records optical spectra with higher accuracy than absorption spectroscopy and allows one to extract the optical constants of the studied structures. The ellipsometric parameters Ψ (ellipsometric reflection) and Δ (ellipsometric phase) were measured using a J. A. Woollam ellipsometer in the 300−1600 nm wavelength range. The ellipsometric parameters Ψ and ∆ are related to the sample amplitude reflections as $\tan(\Psi)\exp\left( i\Delta\right)={r_{p}}/{r_{s}}$, where *r_p_* and *r_s_* are the amplitude reflection coefficients for *p*- and *s*-polarized light, respectively. Both functions (Ψ and Δ) strongly depend on the optical properties of investigated samples and can be used to extract optical constants of investigated layers. To determine the complex refractive index *N = n + ik* of the studied GO/CS membranes (with or without extracted gold), ellipsometric functions Ψ and Δ were experimentally measured and fitted using a Fresnel model.


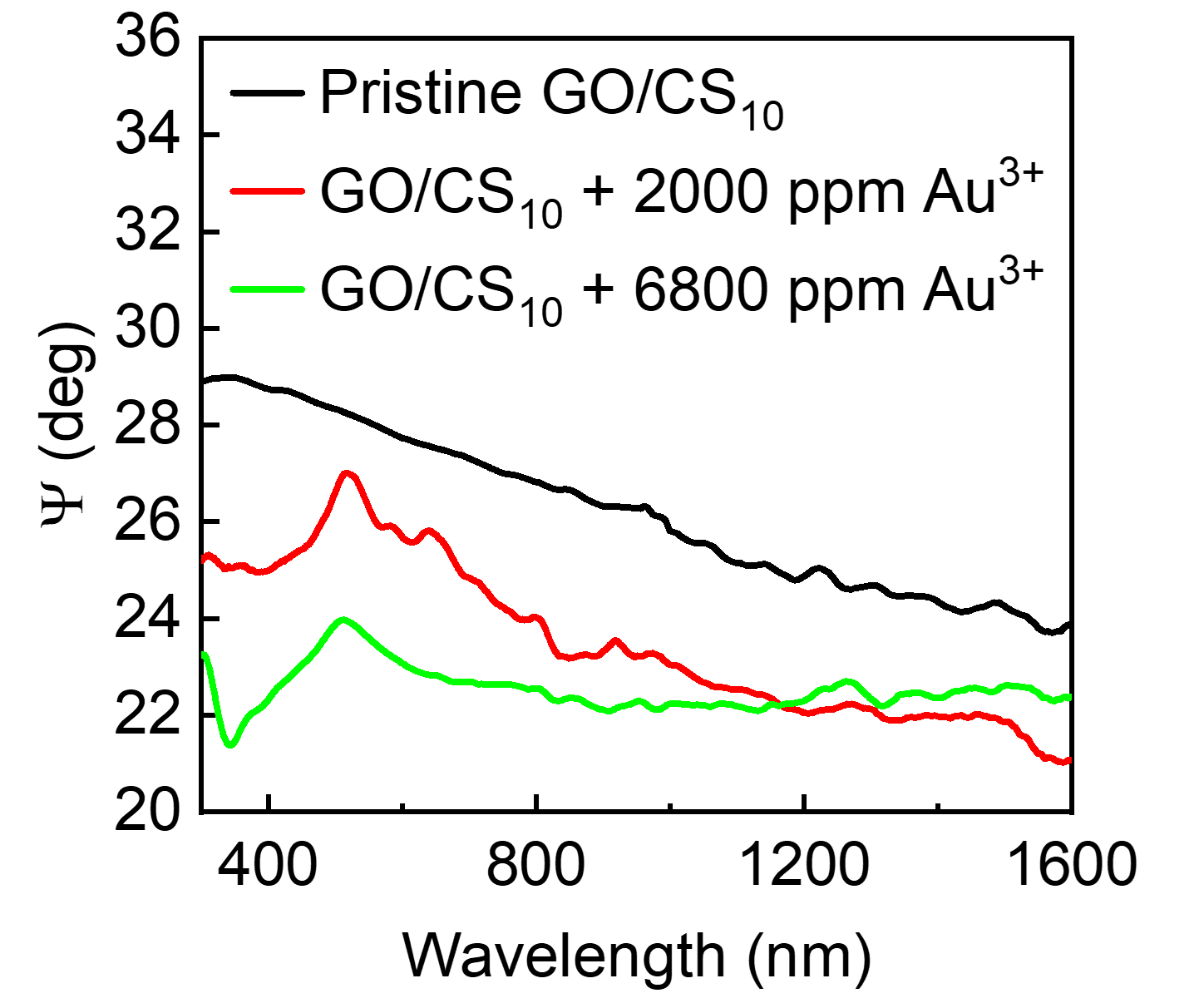


**Supplementary Fig.6** The amplitude spectroscopic parameter Ψ as a function of wavelength measured at the incident angle of 74° for GO/CS membranes subject to different concentrations of gold.

**Supplementary Table 1.** Au^3+^ extraction capacity and equilibrium time of gold absorbents.

| Reference | Gold absorbent | Types of gold ion | Gold ion concentration (ppm) | Equilibrium time (min) | Extraction capacity (mg/g) |
| --- | --- | --- | --- | --- | --- |
| [1] | Thiol-terminated HBPEI functionalized rice straw | Au^3+^ | 500 | 120 | 450 |
| [2] | Ca^II^Cu^II^_6_[(*S,S*)-me-thox]_3_(OH)_2_(H_2_O) |  | 600 | 60 | 598 |
| [3] | cross-linked lignophenol | [AuCl_4_]^-^ | 80 | 1440 | 315 |
| [4] | l-lysine crosslinked chitosan |  | 400 | 240 | 70.3 |
| [5] | NH_2_-MCM-41 |  | 600 | 20 | 226 |
| [6] | Barley straw carbon |  | 100 | 1440 | 256 |
| [7] | taurine modified cellulose |  | 100 | 1440 | 32 |
| [8] | UiO-66-TU |  | 800 | 180 | 326 |
| [9] | UiO-66-NH_2_ |  | 100 | 25 | 100 |
| [10] | PAF-1-thiourea |  | 500 | 120 | 2629.9 |
| [11] | COP-180 |  | 20 | 30 | 100 |
| [11] | COP-180 |  | 3000 | 30 | 1620 |
| [12] | Amyloid-like protein membrane |  | 197 | 1440 | 500 |
| [13] | COP-224 |  | 30 | 1320 | 50 |
| [14] | MoS_2_ nanoflakes |  | 450 | 40 | 1133 |
| [15] | UiO-66-TA |  | 150 | 500 | 260 |
| [15] | UiO-66-TA |  | 900 | 500 | 374 |
| [16] | CNT-MoS_2_ |  | 100 | 100 | 1000 |
| [16] | CNT-MoS_2_ |  | 1000 | 100 | 2495 |
| [17] | rGO nanosheets |  | 10 | 1800 | 1850 |
| this work | GO/CS membrane | Au^3+^ | 15 | 10 | 52 |
|  |  |  | 107 | 10 | 315 |
|  |  |  | 1100 | 10 | 3989 |
|  |  |  | 2267 | 10 | 7830 |

**Reference**

[1] J. Wang, J. Li, J. Wei, *Journal of Materials Chemistry A* **2015**, *3* (35), 18163.

[2] M. Mon, J. Ferrando-Soria, T. Grancha, F. R. Fortea-Perez, J. Gascon, A. Leyva-Perez, D. Armentano, E. Pardo, *Journal of the American Chemical Society* **2016**, *138* (25), 7864.

[3] D. Parajuli, C. R. Adhikari, M. Kuriyama, H. Kawakita, K. Ohto, K. Inoue, M. Funaoka, *Industrial & engineering chemistry research* **2006**, *45* (1), 8.

[4] K. Fujiwara, A. Ramesh, T. Maki, H. Hasegawa, K. Ueda, *Journal of hazardous materials* **2007**, *146* (1-2), 39.

[5] K. F. Lam, C. M. Fong, K. L. Yeung, G. Mckay, *Chemical Engineering Journal* **2008**, *145* (2), 185.

[6] R. Chand, T. Watari, K. Inoue, H. Kawakita, H. N. Luitel, D. Parajuli, T. Torikai, M. Yada, *Minerals Engineering* **2009**, *22* (15), 1277.

[7] A. D. Dwivedi, S. P. Dubey, S. Hokkanen, R. N. Fallah, M. Sillanpää, *Chemical Engineering Journal* **2014**, *255*, 97.

[8] C. Wu, X. Zhu, Z. Wang, J. Yang, Y. Li, J. Gu, *Industrial & Engineering Chemistry Research* **2017**, *56* (47), 13975.

[9] S. Lin, D. H. K. Reddy, J. K. Bediako, M.-H. Song, W. Wei, J.-A. Kim, Y.-S. Yun, *Journal of Materials Chemistry A* **2017**, *5* (26), 13557.

[10] T. Ma, R. Zhao, Z. Li, X. Jing, M. Faheem, J. Song, Y. Tian, X. Lv, Q. Shu, G. Zhu, *ACS applied materials & interfaces* **2020**, *12* (27), 30474.

[11] Y. Hong, D. Thirion, S. Subramanian, M. Yoo, H. Choi, H. Y. Kim, J. F. Stoddart, C. T. Yavuz, *Proceedings of the National Academy of Sciences* **2020**, *117* (28), 16174.

[12] F. Yang, Z. Yan, J. Zhao, S. Miao, D. Wang, P. Yang, *Journal of Materials Chemistry A* **2020**, *8* (6), 3438.

[13] T. S. Nguyen, Y. Hong, N. A. Dogan, C. T. Yavuz, *Chemistry of Materials* **2020**, *32* (12), 5343.

[14] B. Feng, C. Yao, S. Chen, R. Luo, S. Liu, S. Tong, *Chemical engineering journal* **2018**, *350*, 692.

[15] C. Wang, G. Lin, J. Zhao, S. Wang, L. Zhang, Y. Xi, X. Li, Y. Ying, *Chemical Engineering Journal* **2020**, *380*, 122511.

[16] F. Liu, S. You, Z. Wang, Y. Liu, *ACS ES&T Engineering* **2021**, *1* (9), 1342.

[17] F. Li, J. Zhu, P. Sun, M. Zhang, Z. Li, D. Xu, X. Gong, X. Zou, A. Geim, Y. Su, *Nature communications* **2022**, *13* (1), 1.
